# Validity of the Vernier Go Direct Force Plate for Measuring Vertical Jump Performance

**DOI:** 10.3390/s26144481

**Published:** 2026-07-15

**Authors:** Xin-Mei Lee, Chin-Yi Gu, Li-I Wang, Wei-Han Chen

**Affiliations:** 1 Department of Physical Education and Kinesiology, National Dong Hwa University, Hualien 974301, Taiwan; 611389001@gms.ndhu.edu.tw (X.-M.L.); gucyyy@gms.ndhu.edu.tw (C.-Y.G.); tennis01@gms.ndhu.edu.tw (L.-I.W.); 2 Sports Science and Technology Center, National Dong Hwa University, Hualien 974301, Taiwan

**Keywords:** countermovement jump, squat jump, athlete, health, technology

## Abstract

The purpose of this study was to evaluate the validity of the Vernier Go Direct force plate against a laboratory-grade AMTI force plate during squat jump (SJ) and countermovement jump (CMJ) assessments. Forty physically active university students (20 males and 20 females) performed both jump tests while vertical ground reaction force was recorded simultaneously by both systems. A total of 28 force-time variables were analyzed (9 from SJ and 19 from CMJ). For SJ, all parameters demonstrated excellent agreement (ICC = 0.986–0.997; CCC = 0.986–0.997) with small biases ranging from −4.0% to 3.6%. For CMJ, the parameters also showed good to excellent agreement (ICC = 0.846–0.999; CCC = 0.845–0.999) and minimal biases (−3.1% to 3.3%). These findings support the validity of the Vernier Go Direct force plate for measuring vertical jump performance and can serve as a cost-effective alternative for dynamic strength assessment, applied sports science research, and physical education settings.

## 1. Introduction

In sport science, performance assessments are essential for monitoring athletes’ physical condition, evaluating training adaptations, guiding competition preparation, and reducing injury risk [1,2]. Among various measurement tools, the force plate is one of the most widely used in sport science research [3]. Force plates provide real-time analysis of vertical ground reaction force (vGRF) characteristics, offering valuable insights into an individual’s neuromuscular function and physical condition. Force plates thus serve as critical instruments for coaches, medical professionals, and physical education teachers in assessing performance and informing training, rehabilitation, and teaching strategies, ultimately supporting tailored exercise prescriptions to optimize outcomes [3,4,5].

The squat jump (SJ) and countermovement jump (CMJ) are among the most frequently used force plate assessments for evaluating lower-limb neuromuscular performance in athletes [3]. Although both tests assess vertical jump ability, they reflect different physiological and mechanical characteristics. The SJ primarily evaluates concentric force-generating capacity, whereas the CMJ incorporates the stretch–shortening cycle (SSC), providing additional information regarding neuromuscular function and movement efficiency [3,6]. Force plate analyses of vertical jump tasks provide a wide range of temporal, kinetic, and performance-related variables that can be used to evaluate lower-limb neuromuscular function and movement performance [3,6,7]. Common temporal and performance-related variables include unweighting phase duration (T_unweighting_), braking phase duration (T_braking_), amortization phase duration (T_amortization_), propulsion phase duration (T_propulsion_), jump height (JH), and reactive strength index modified (RSI_mod_) [5,8,9]. Kinetic variables include net impulse, peak force, rate of force development (RFD), peak power, work, and leg stiffness [4,5,10]. Collectively, these variables provide valuable information regarding lower-limb force production, movement strategy, neuromuscular performance, and SSC function, making them widely used in sports performance assessment, fatigue monitoring, and rehabilitation settings [3,4,5,10].

Traditional laboratory-grade force plate systems are generally expensive, require dedicated laboratory space, and are less accessible for routine field-based testing. To address these limitations, several portable and wireless force plate systems have been developed and validated, including the PASCO PS-2142 (PASCO Scientific, Roseville, CA, USA), Hawkin Dynamics (Hawkin Dynamics Inc., Westbrook, ME, USA), Kunwei KWYP-FP6035 (Changzhou Kunwei Sensing Technology Co., Ltd., Changzhou, China), and Kinvent KForce (KINVENT Biomecanique, Montpellier, France) [11,12,13,14,15]. Previous validation studies have demonstrated acceptable agreement for selected variables, including JH, peak force, power, impulse, flight time, or RSI_mod_ [11,12,13,14,15]. However, comprehensive validation of phase-specific temporal variables (e.g., unweighting, braking, amortization, and propulsion phase durations) and additional force plate-derived variables (e.g., RFD, work) across both SJ and CMJ tasks remains limited. Therefore, further validation studies are required to determine whether portable force plate systems can accurately quantify the comprehensive range of temporal and kinetic variables commonly used in vertical jump assessments.

The Vernier Go Direct Force Plate is a portable wireless force plate system originally developed for educational and field-based applications. Compared with previously validated portable systems, the device offers a low-cost and user-friendly alternative that may facilitate wider adoption of force plate testing in educational and applied sport settings. However, despite its growing use in educational environments, its measurement validity has not yet been established against a laboratory-grade reference system. AMTI force plates are widely used in biomechanics laboratories and have been extensively adopted as criterion-reference systems in previous force plate validation studies because of their high accuracy, stability, and measurement reliability [12,13,14]. Therefore, the purpose of this study was to examine the validity of the Vernier Go Direct Force Plate by comparing its measurements with those obtained from an AMTI force plate. It was hypothesized that the Vernier Go Direct Force Plate would demonstrate excellent agreement and strong correlations with the AMTI force plate across a comprehensive set of temporal and kinetic variables obtained during SJ and CMJ assessments.

## 2. Materials and Methods

### 2.1. Participants

Forty physically active university students (20 males and 20 females; mean ± SD: age 23.2 ± 1.8 years, height 169.1 ± 8.8 cm, body mass 67.3 ± 11.1 kg) participated in the study. The sample size was determined with reference to previous force plate validation studies, which typically included 20–30 participants [12,14]. Participants were either athletes from the university’s sports teams or students from the Department of Physical Education and Kinesiology. They were selected because they were familiar with vertical jump testing and capable of performing SJ and CMJ tasks consistently, thereby reducing movement variability during testing.

Male participants (*n* = 20) had a mean age of 22.9 ± 1.6 years, height of 175.8 ± 5.0 cm, and body mass of 74.9 ± 7.6 kg. Female participants (*n* = 20) had a mean age of 23.6 ± 1.9 years, height of 162.4 ± 6.2 cm, and body mass of 59.7 ± 8.7 kg.

All participants were in good health, had normal visual ability, and reported no history of lower-limb bone or ligament surgery within the previous six months. Written informed consent was obtained from all participants before data collection. This study was conducted in accordance with the Declaration of Helsinki and approved by the Human Research Ethics Committee (NCKU HREC-E-114-1029-2).

### 2.2. Procedure

Prior to testing, participants completed a self-directed warm-up consisting of light aerobic activity and dynamic stretching for 5–10 min, followed by 2 min of seated rest. Participants completed the CMJ condition before the SJ condition. This order was selected because the CMJ represents a more natural jumping movement and allowed participants to become familiar with the testing environment before performing the more technically constrained SJ task. For each jump condition, participants performed two familiarization trials followed by three recorded trials. A 30-s rest interval was provided between consecutive trials to minimize the influence of fatigue. A 3-min rest interval was provided between jump conditions to minimize the influence of fatigue between testing conditions.

For the CMJ, participants stood upright with their hands placed on their hips and eyes facing forward. Upon the verbal command “jump”, participants performed a rapid downward countermovement followed immediately by a maximal vertical jump. For the SJ, participants stood on the force plate with their hands on their hips and eyes facing forward. Starting from a static half-squat position, participants were instructed to perform a maximal vertical jump upon hearing the verbal command without any preparatory countermovement, thereby minimizing the contribution of the stretch–shortening cycle. The vGRF-time curve was inspected immediately after each SJ trial. Trials exhibiting an initial decrease in vGRF exceeding 2.5% of body weight before push-off, indicative of a countermovement, were deemed invalid and repeated. For both the CMJ and SJ, squat depth was self-selected.

### 2.3. Data Collection

**Figure 1:**
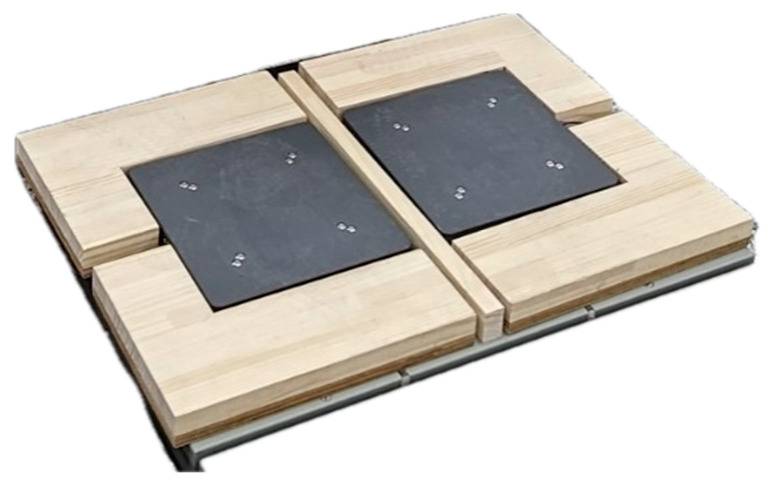
Example set-up for data collection.

A triaxial force plate (AMTI 6886, AMTI Inc., Watertown, MA, USA; 60 × 90 cm; 1000 Hz) was securely mounted to the laboratory floor and served as the criterion reference system for all measurements. Two wireless force plates (Vernier Go Direct; Vernier Science Education, Beaverton, OR, USA; 31.5 × 31.5 cm; 1000 Hz) were placed centrally on top of the AMTI force plate without rigid mechanical fixation, consistent with a previous validation study [14], enabling simultaneous measurement of vGRF during each jump trial. Prior to data collection, the alignment and stability of the stacked force plate configuration were verified. Pilot testing confirmed that no visible displacement or slippage of the Vernier force plates occurred during take-off or landing. A surrounding wooden platform of the same height was constructed around the force plates to provide a stable landing surface and ensure participant safety during testing. The surrounding platform was positioned more than 0.5 cm from the force plates to prevent physical contact between the structures during testing and to minimize potential mechanical interference with force measurements (Figure 1). The AMTI force plate was connected in sequence to the AMTI signal amplifier, a Qualisys 64-channel A/D box, and a laptop computer. All cables were arranged to avoid interference on the measurement plane. The vGRF data was collected using Qualisys Track Manager (QTM) version 2.8 (Qualisys AB, Gothenburg, Sweden). The Vernier Go Direct force plates were connected to a laptop computer via the original transmission cable, and data were acquired using Vernier Graphical Analysis software. Both force plate systems sampled data at 1000 Hz.

### 2.4. Data Processing

ProGRF-SJ Analysis 1.5 (East coast sport science studio, Taiwan) and ProGRF-CMJ Analysis 1.13 (East coast sport science studio, Taiwan) software were used to analyze the vGRF data of SJ and CMJ actions. Force-time data were analyzed using the default processing procedures implemented within the ProGRF-SJ Analysis 1.5 and ProGRF-CMJ Analysis 1.13. No additional filtering procedure was applied prior to analysis [14,16]. The SJ analysis parameters included total movement time (T_total_), JH based on flight time (JH_flight-time_), JH based on impulse (JH_impulse_), RSI_mod_, net impulse, peak propulsive force (F_propulsive-peak_), RFD in propulsion (RFD_propulsion_), peak propulsive power (P_propulsive-peak)_, and propulsive work (W_propulsive_). The CMJ analysis parameters included T_unweighting_, T_braking_, T_propulsion_, T_amortization_, T_total_, JH_flight-time_, JH_impulse_, RSI_mod_, net impulse, peak unweighting force (F_unweighting-peak_), Force at zero velocity (F_zero-velocity_), RFD in braking (RFD_braking_), peak braking power (P_braking-peak_), braking work (W_braking_), F_propulsive-peak_, RFD_propulsion_, P_propulsive-peak_, W_propulsive_, and leg stiffness. Selected force-time variable calculations included the following:

1. Unweighting phase duration (T_unweighting_): The time from the start of movement (vGRF decreases by more than 2.5% of body weight [17]) to the end of the unweighting phase (vGRF ≥ body weight). Body weight was calculated as the mean vGRF during the initial 0.1 s of data collection based on the default setting of the ProGRF software. Participants stood quietly on the force plate before initiating the jump.
2. Braking phase duration (T_braking_): The time from the end of the unweighting phase to the lowest point of the center of mass.
3. Amortization phase duration (T_amortization_): The time spanning the 1 cm vertical displacement before and after the lowest point of the center of mass, which occurs at the transition between the braking and propulsion phases [8].
4. Propulsion phase duration (T_propulsion_): The time from the lowest point of the center of mass to the moment of take-off (net vGRF < 10 N [18]).
5. Total movement time (T_total_): The total duration from beginning to take-off.
6. Jump height based on flight time (JH_flight-time_): JH = (t^2^ × g) ÷ 8, where *t* is flight time and *g* is the acceleration due to gravity.
7. Jump height based on impulse (JH_impulse_): It was calculated using the impulse–momentum method. Take-off velocity was determined from the net vertical impulse. JH_impulse_ = V_to_^2^ ÷ 2 g, where *V_to_* is the take-off velocity and *g* is the acceleration due to gravity.
8. Reactive strength index modified (RSI_mod_): It was calculated by dividing the JH_flight-time_ by the T_total_ [9].
9. Net impulse: The integral of the net vGRF with time.
10. Peak unweighting force (F_unweighting-peak_): The minimum vGRF observed during the unweighting phase, representing the lowest force value reached during the unweighting phase.
11. Force at zero velocity (F_zero-velocity_): The vGRF measured at the end of the braking phase, corresponding to the instant at which the center-of-mass velocity equals zero.
12. Rate of force development in braking (RFD_braking_): It was calculated by dividing the F_zero-velocity_ by the T_braking_.
13. Peak braking power (P_braking-peak_): The minimum power generated during the braking phase.
14. Braking work (W_braking_): The total amount of work done in the braking phase.
15. Peak propulsive force (F_propulsive-peak_): The maximum vGRF measured during the jump.
16. Rate of force development in propulsion (RFD_propulsion_): It was calculated by dividing the F_propulsive-peak_ by the time from end of the unweighting phase to F_propulsive-peak_.
17. Peak propulsive power (P_propulsive-peak_): The maximum power generated during the propulsion phase.
18. Propulsive work (W_propulsive_): The total amount of work done during the propulsion phase.
19. Leg stiffness: Calculated as the ratio of F_zero-velocity_ to the vertical displacement during the unweighting phase.

### 2.5. Statistical Analysis

SPSS Statistics 29.0 was used for statistical analysis. Intraclass correlation coefficients (ICC [3,1], absolute agreement, single measurement) [19] and Lin’s concordance correlation coefficients (CCC) were calculated to assess the agreement between the Vernier Go Direct and AMTI force plate systems. ICC values were interpreted as poor (ICC < 0.50), moderate (ICC = 0.50–0.75), good (ICC = 0.75–0.90), and excellent (ICC > 0.90) [19]. Pearson’s correlation coefficients (*r*) and ordinary least squares (OLS) regression analyses were performed to evaluate the linear association between the two force plate systems. Regression equations, slope coefficients, and coefficients of determination (*R*^2^) are presented. Pearson’s correlation coefficients were interpreted as negligible (0.00–0.10), weak (0.10–0.39), moderate (0.40–0.69), strong (0.70–0.89), and very strong (0.90–1.00) [20]. Cohen’s *d* effect sizes (ES) were calculated to quantify the magnitude of differences between the two force plate systems, with values of 0.20, 0.50, and 0.80 interpreted as small, medium, and large, respectively [21]. Mean difference, bias, bias percentage (bias/mean × 100%), and limits of agreement (LoA = bias ± 1.96 SD) were calculated using the Bland–Altman method [22]. The statistical significance was set at the α = 0.05 level.

## 3. Results

**Figure 2:**
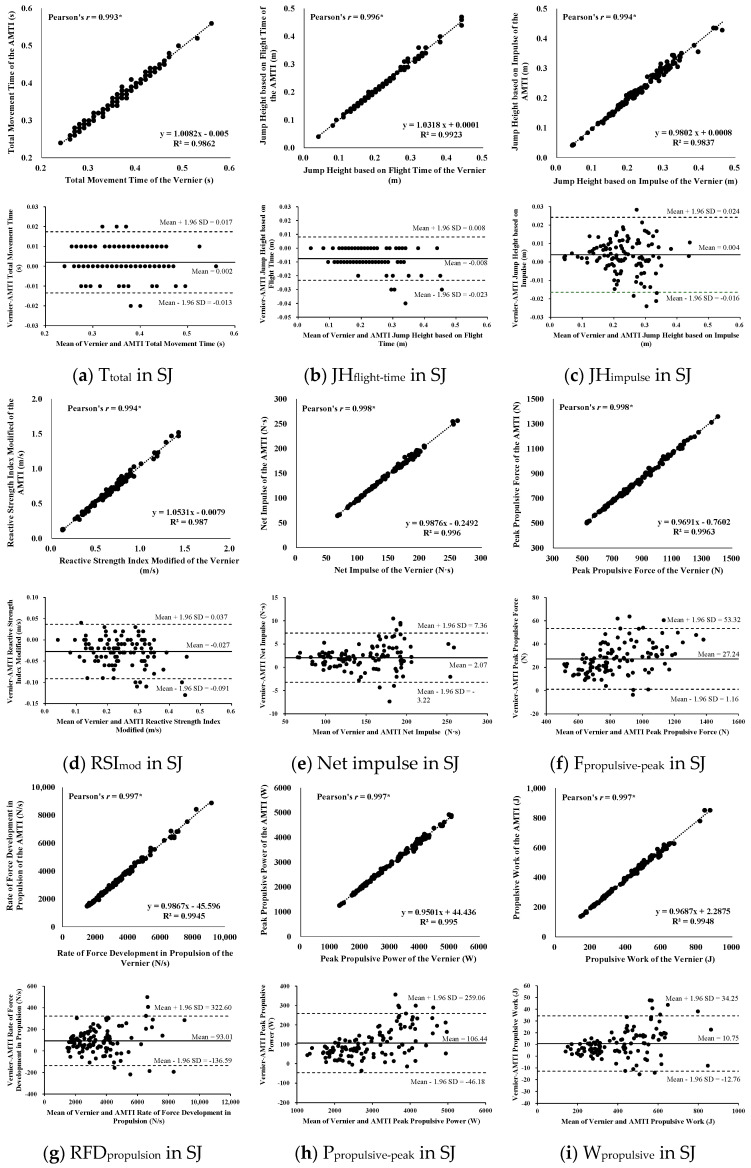
Scatter and Bland–Altman plots for squat jump variables. * *p* < 0.05; T_total_ = total movement time; JH_flight-time_ = jump height based on flight time; JH_impulse_ = jump height based on impulse; RSI_mod_ = reactive strength index modified; F_propulsive-peak_ = peak propulsive force; RFD_propulsion_ = rate of force development in propulsion; P_propulsive-peak_ = peak propulsive power; W_propulsive_ = propulsive work.

The validity results for the SJ variables are presented in Table 1 and Figure 2. Most SJ variables demonstrated excellent agreement between the Vernier Go Direct and AMTI force plate systems, with ICC and CCC values ranging from 0.986 to 0.997. However, according to the 95% confidence intervals, F_propulsive-peak_ demonstrated poor to excellent agreement and P_propulsive-peak_ demonstrated good to excellent agreement. Bias percentages ranged from −4.0% to 3.6%, and effect sizes were trivial to small.

**Figure 3:**
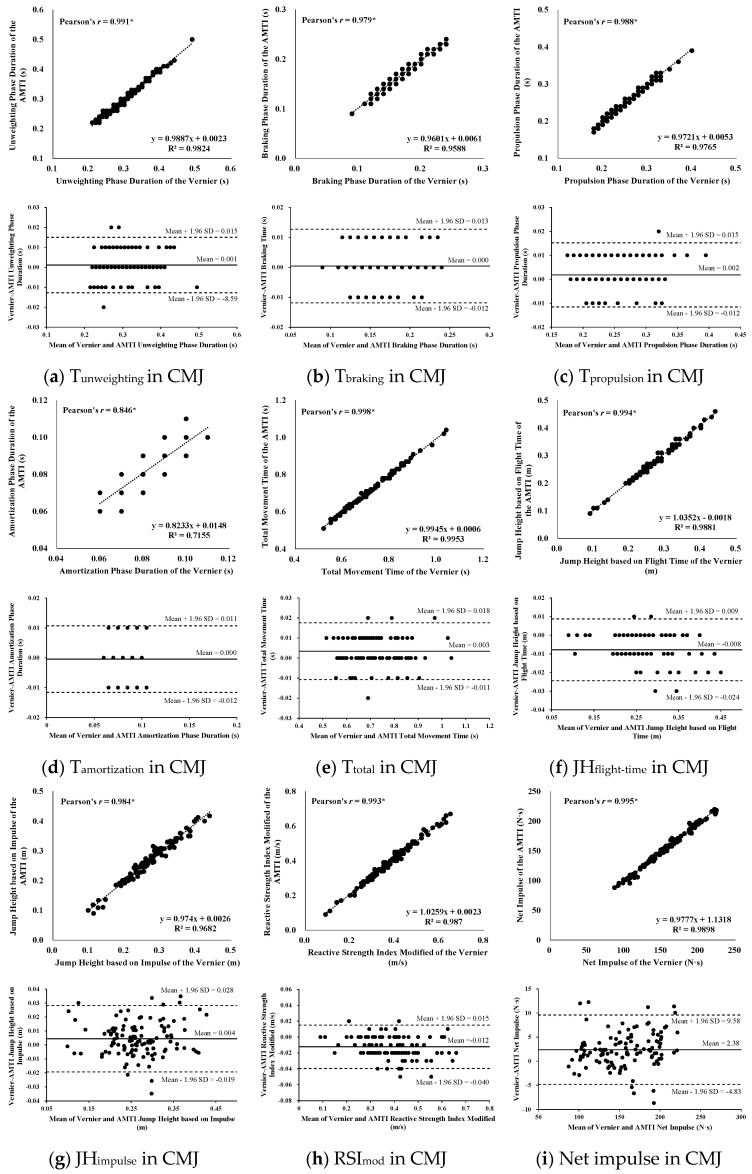
Scatter and Bland–Altman plots for countermovement jump variables. * *p* < 0.05; T_unweighting_ = unweighting phase duration; T_braking_ = braking phase duration; T_propulsion_ = propulsion phase duration; T_amortization_ = amortization phase duration; T_total_ = total movement time; JH_flight-time_ = jump height based on flight time; JH_impulse_ = jump height based on impulse; RSI_mod_ = reactive strength index modified.

**Figure 4:**
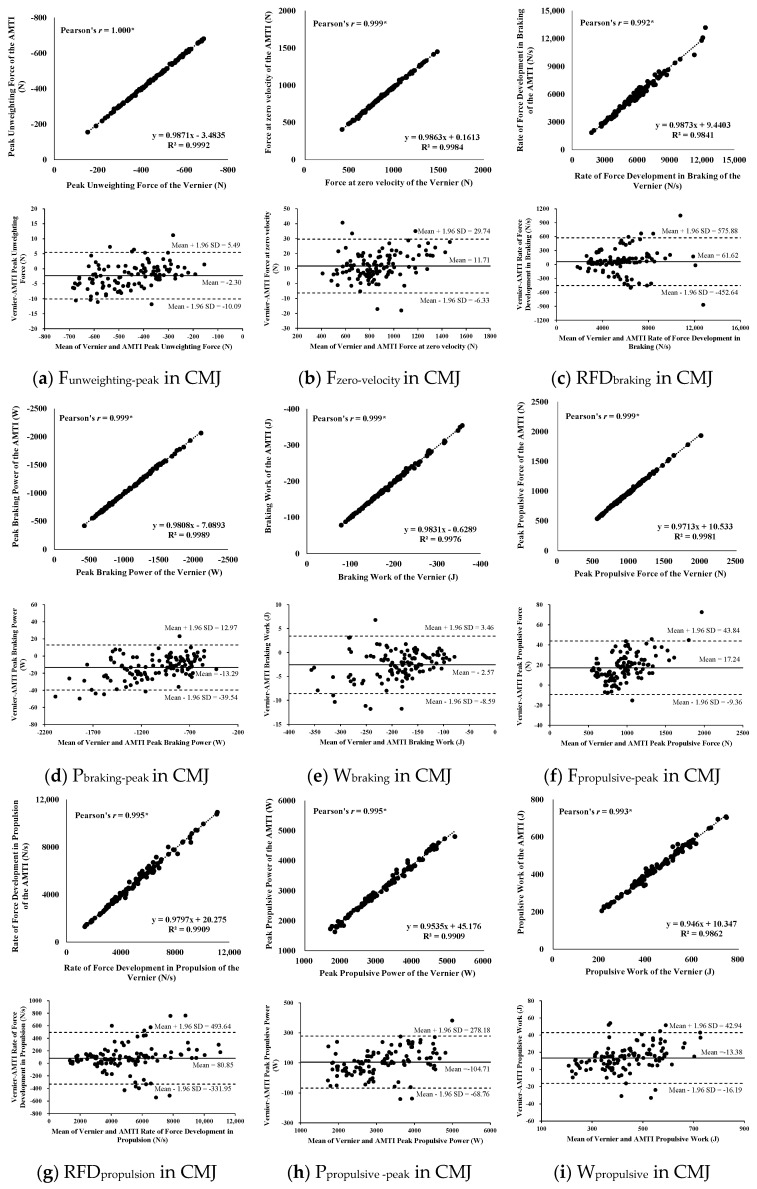
Scatter and Bland–Altman plots for countermovement jump kinetic variables. * *p* < 0.05; F_unweighting-peak_ = peak unweighting force; F_zero-velocity_ = force at zero velocity; RFD_braking_ = rate of force development in braking; P_braking -peak_ = peak braking power; W_braking_ = braking work; F_propulsive-peak_ = peak propulsive force; RFD_propulsion_ = rate of force development in propulsion; P_propulsive-peak_ = peak propulsive power; W_propulsive_ = propulsive work.

**Figure 5:**
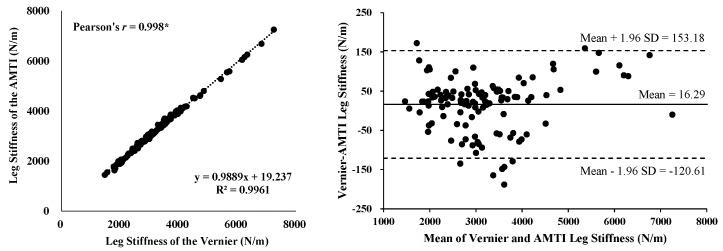
Scatter and Bland–Altman plots for countermovement jump leg stiffness. * *p* < 0.05.

The validity results for the CMJ variables are presented in Table 2 and Figure 3, Figure 4 and Figure 5. Most CMJ variables demonstrated excellent agreement between the two force plate systems, with ICC and CCC values ranging from 0.979 to 0.999. However, T_amortization_ and P_propulsive-peak_ demonstrated good to excellent agreement according to their 95% confidence interval. Bias percentages ranged from −3.1% to 3.3%. All effect sizes were trivial to small.

## 4. Discussion

The main findings of this study indicate that the Vernier Go Direct and AMTI force plate systems demonstrated a high level of agreement across all measured parameters during both the SJ and CMJ. This was consistently supported by the combined results of the ICC, CCC, OLS regression, bias, and Bland–Altman analyses, indicating minimal systematic differences between the two systems. Amortization phase duration remained the only variable showing comparatively lower agreement (ICC = 0.846; CCC = 0.845), although the bias remained minimal (0.5%). Collectively, these findings support the validity of the Vernier Go Direct force plate as a practical alternative to the laboratory-standard AMTI system for assessing vertical jump performance.

Researchers have examined several commercially available portable and wireless force plate systems and reported good validity in vertical jump assessment [11,12,13,14,15]; however, the validated parameters in those studies were often limited in scope compared with the present findings. For instance, Peterson Silveira et al. [11] verified the vertical impulse (*r* = 0.989), peak force (*r* = 0.996), and time to peak force (*r* = 0.997) of the Pasco PS-2142 system against a Kistler 9287 force plate. Mao et al. [14] reported excellent agreement (ICC = 0.955–1.000) for body weight, JH, F_propulsive-peak_, P_propulsive-peak_, take-off velocity, T_total_, T_propulsion_, flight time, propulsive impulse, and RSI_mod_ between the Kunwei KWYP-FP6035 and the Kistler 9287CA force plate. Likewise, very strong correlation (*r* = 0.841–1.000) and negligible bias were observed between the Hawkin Dynamics and AMTI force plates during CMJ assessments for RSI, RSI_mod_, JH, flight time, T_total,_ and leg stiffness and for force, velocity, power, and impulse across both propulsive and braking phases [12,13]. The good to excellent agreement observed in the present study (ICC = 0.846–0.999) is consistent with these previous validation studies, further supporting the validity of portable force plate systems for vertical jump assessment. Despite differences in hardware design and signal processing algorithms among the tested devices, all studies reported strong agreement with laboratory-grade force plates, suggesting that portable force plate technology can provide reliable measurements in both research and applied settings. Furthermore, whereas previous studies primarily focused on jump outcome variables (e.g., JH, flight time, impulse, peak force, power, RSI_mod_), the present study additionally examined agreement across a wider range of force-time-derived variables. This broader evaluation provides further evidence regarding the capability of portable force plates to quantify multiple biomechanical characteristics of jumping performance beyond conventional outcome measures.

Amortization phase duration (T_amortization_) was the only parameter that demonstrated good agreement (ICC = 0.846), although the bias remained minimal (0.5%). This finding suggests that T_amortization_ is more sensitive to subtle inter-device variations than other variables. In this study, the mean T_amortization_ was 0.08 ± 0.01 s, representing an extremely short transition period around the lowest position of the center of mass where vertical motion reverses from braking to propulsion. Because this interval is so brief, even minor discrepancies in detecting the exact inflection point of the vGRF–time curve can lead to noticeable differences between systems. Such discrepancies may result from variations in platform stiffness, sensor response characteristics, vertical displacement computation, or the serial setup used in the present study, in which the Vernier force plate was positioned on top of the AMTI force plate. This configuration may have introduced subtle differences in load transmission and interface compliance between systems. Nevertheless, the observed bias was minimal (0.5%), suggesting that the portable system still provides adequate precision for practical assessments of phase transition timing in vertical jump testing.

Several limitations should be acknowledged. First, the participants were physically active university students familiar with vertical jump testing; therefore, the findings may not be generalizable to other populations. Second, only SJ and CMJ tasks were evaluated, and the validity of the Vernier Go Direct force plate during other athletic movements remains unknown. Third, although no visible displacement or slippage was observed during pilot testing, the two force plate systems were tested simultaneously using a stacked configuration without additional mechanical fixation. Therefore, a small degree of mechanical interaction or undetected relative movement between devices cannot be completely excluded. Future studies should examine the validity and reliability of the Vernier Go Direct force plate across different populations and movement tasks.

## 5. Conclusions

In conclusion, the findings of this study demonstrated good to excellent agreement and consistent linear relationships between the Vernier Go Direct and AMTI force plates in measuring both temporal and kinetic variables during SJ and CMJ, with only minimal deviations observed across variables. These findings support the validity of the Vernier Go Direct force plate for assessing vertical jump performance. Accordingly, it can serve as a reliable and cost-effective alternative to laboratory-grade systems for assessing dynamic lower-limb neuromuscular performance, supporting applied sports science research, and facilitating physical education.

## Figures and Tables

**Table 1:** Comparison in squat jump variables between Vernier Go Direct and AMTI force plates.

	Vernier	AMTI								Bland–Altman				
Metric	M ± SD	M ± SD	ICC	95% CI	CCC	95% CI	*r*	Slope	ES	Bias	Bias%	SD Bias	LoA	LoA
T_total_ (s)	0.361 ± 0.066	0.359 ± 0.067	0.993 *	(0.989–0.995)	0.993	(0.989–0.995)	0.993 *	1.0082	0.030	0.002	0.6	0.008	0.017	−0.013
JH_flight-time_ (m)	0.236 ± 0.083	0.244 ± 0.086	0.992 *	(0.942–0.997)	0.992	(0.988–0.994)	0.996 *	1.0318	−0.089	−0.008	−3.1	0.008	0.008	−0.023
JH_impulse_ (m)	0.237 ± 0.081	0.239 ± 0.080	0.991 *	(0.983–0.993)	0.991	(0.986–0.993)	0.992 *	0.9802	−0.026	0.004	1.7	0.010	0.024	−0.016
RSI_mod_ (m/s)	0.662 ± 0.247	0.689 ± 0.262	0.986 *	(0.932–0.995)	0.986	(0.981–0.990)	0.994 *	1.0531	−0.107	−0.027	−4.0	0.033	0.037	−0.091
Net impulse (N·s)	147.3 ± 42.4	145.2 ± 41.9	0.997 *	(0.987–0.999)	0.997	(0.995–0.998)	0.998 *	0.9876	0.049	2.1	1.4	2.7	7.4	−3.2
F_propulsive-peak_ (N)	856.4 ± 200.3	829.2 ± 194.5	0.988 *	(0.417–0.997)	0.988	(0.984–0.991)	0.998 *	0.9691	0.138	27.2	3.2	13.3	53.3	1.2
RFD_propulsion_ (N/s)	3551.8 ± 1576.1	3458.8 ± 1559.3	0.995 *	(0.980–0.998)	0.995	(0.994–0.997)	0.997 *	0.9867	0.059	93.0	2.7	117.1	322.6	−136.6
P_propulsive-peak_ (W)	3021.6 ± 926.4	2915.1 ± 882.4	0.989 *	(0.783–0.997)	0.989	(0.986–0.992)	0.997 *	0.9501	0.118	106.4	3.6	77.9	259.1	−46.2
W_propulsive_ (J)	416.2 ± 156.7	405.5 ± 152.2	0.995 *	(0.967–0.998)	0.995	(0.992–0.996)	0.997 *	0.9687	0.070	10.7	2.6	12.0	34.3	−12.8

* *p* < 0.05; T_total_ = total movement time; JH_flight-time_ = jump height based on flight time; JH_impulse_ = jump height based on impulse; RSI_mod_ = reactive strength index modified; F_propulsive-peak_ = peak propulsive force; RFD_propulsion_ = rate of force development in propulsion; P_propulsive-peak_ = peak propulsive power; W_propulsive_ = propulsive work.

**Table 2:** Comparison in countermovement jump variables between Vernier Go Direct and AMTI force plates.

	Vernier	AMTI								Bland–Altman				
Metric	M ± SD	M ± SD	ICC	(95% CI)	CCC	(95% CI)	*r*	Slope	ES	Bias	Bias%	SD Bias	LoA	LoA
T_unweighting_ (s)	0.305 ± 0.054	0.303 ± 0.053	0.991 *	(0.987–0.994)	0.991	(0.987–0.994)	0.991 *	0.9887	0.021	0.001	0.4	0.007	0.015	−0.013
T_braking_ (s)	0.163 ± 0.031	0.163 ± 0.030	0.979 *	(0.970–0.985)	0.979	(0.970–0.985)	0.979 *	0.9601	0.014	0.000	0.3	0.006	0.013	−0.012
T_propulsion_ (s)	0.256 ± 0.045	0.254 ± 0.044	0.987 *	(0.981–0.991)	0.987	(0.982–0.991)	0.988 *	0.9721	0.041	0.002	0.7	0.007	0.015	−0.012
T_amortization_ (s)	0.081 ± 0.010	0.082 ± 0.010	0.846 *	(0.785–0.891)	0.845	(0.783–0.890)	0.846 *	0.8233	−0.043	0.000	−0.5	0.006	0.011	−0.012
T_total_ (s)	0.724 ± 0.106	0.720 ± 0.016	0.997 *	(0.994–0.998)	0.997	(0.996–0.998)	0.998 *	0.9945	0.032	0.003	0.5	0.007	0.018	−0.011
JH_flight-time_ (m)	0.274 ± 0.071	0.281 ± 0.074	0.988 *	(0.921–0.995)	0.987	(0.983–0.991)	0.994 *	1.0352	−0.108	−0.008	−2.8	0.008	0.009	−0.024
JH_impulse_ (m)	0.269 ± 0.068	0.264 ± 0.067	0.981 *	(0.968–0.988)	0.982	(0.974–0.987)	0.984 *	0.974	0.065	0.004	1.7	0.012	0.028	−0.019
RSI_mod_ (m/s)	0.378 ± 0.115	0.399 ± 0.119	0.988 *	(0.929–0.995)	0.987	(0.982–0.991)	0.993 *	1.0259	−0.105	−0.012	−3.1	0.014	0.015	−0.040
Net impulse (N·s)	157.3 ± 36.1	154.9 ± 35.5	0.993 *	(0.978–0.996)	0.993	(0.989–0.995)	0.995 *	0.9777	0.066	2.4	1.5	3.700	9.6	−4.8
F_unweighting-peak_ (N)	−449.2 ± 128.5	−446.9 ± 126.9	0.999 *	(0.998–1.000)	0.999	(0.999–1.000)	1.000 *	0.9871	−0.018	−2.3	0.5	4.0	5.5	−10.1
F_zero-velocity_ (N)	868.0 ± 220.4	856.3 ± 217.6	0.998 *	(0.957–0.999)	0.998	(0.997–0.998)	0.999 *	0.9863	0.053	11.71	1.4	9.2	29.7	−6.3
RFD_braking_ (N/s)	5601.9 ± 2080.0	5540.3 ± 2070.2	0.992 *	(0.988–0.994)	0.992	(0.988–0.994)	0.992 *	0.9873	0.030	61.6	0.1	262.4	575.9	−452.6
P_braking-peak_ (W)	−1059.1 ± 354.5	−1045.8 ± 347.9	0.999 *	(0.988–1.000)	0.999	(0.998–0.999)	0.999 *	0.9808	−0.038	−13.3	1.3	13.4	13.0	−39.5
W_braking_ (J)	−189.7 ± 59.8	−187.1 ± 58.8	0.998 *	(0.988–0.999)	0.998	(0.997–0.998)	0.999 *	0.9831	−0.043	−2.6	1.4	3.1	3.5	−8.6
F_propulsive-peak_ (N)	967.9 ± 266.5	950.6 ± 259.1	0.997 *	(0.937–0.999)	0.997	(0.995–0.997)	0.999 *	0.9713	0.066	17.2	1.8	13.6	43.8	−9.4
RFD_propulsion_ (N/s)	4975.3 ± 2197.7	4894.4 ± 2162.8	0.995 *	(0.991–0.997)	0.995	(0.992–0.996)	0.995 *	0.9797	0.037	80.8	1.6	210.6	493.6	−331.9
P_propulsive-peak_ (W)	3221.7 ± 863.0	3117.0 ± 826.6	0.987 *	(0.832–0.996)	0.987	(0.982–0.990)	0.995 *	0.9535	0.124	104.7	3.3	88.5	278.2	−68.8
W_propulsive_ (J)	439.7 ± 121.3	426.3 ± 115.6	0.986 *	(0.919–0.995)	0.986	(0.980–0.990)	0.993 *	0.946	0.113	13.4	3.1	15.1	43.0	−16.2
Leg stiffness (N/m)	3204.8 ± 1115.3	3188.5 ± 1105.1	0.998 *	(0.997–0.999)	0.998	(0.997–0.999)	0.998 *	0.9889	0.015	16.3	0.5	69.8	153.2	−120.6

* *p* < 0.05; T_unweighting_ = unweighting phase duration; T_braking_ = braking phase duration; T_propulsion_ = propulsion phase duration; T_amortization_ = amortization phase duration; T_total_ = total movement time; JH_flight-time_ = jump height based on flight time; JH_impulse_ = jump height based on impulse; RSI_mod_ = reactive strength index modified; F_unweighting-peak_ = peak unweighting force; F_zero-velocity_ = force at zero velocity; RFD_braking_ = rate of force development in braking; P_braking-peak_ = peak braking power; W_braking_ = braking work; F_propulsive-peak_ = peak propulsive force; RFD_propulsion_ = rate of force development in propulsion; P_propulsive-peak_ = peak propulsive power; W_propulsive_ = propulsive work.

## Data Availability

The original contributions presented in this study are included in the article. Further inquiries can be directed to the corresponding author.
